# Antiviral Inflammasomes and How to Find Them

**DOI:** 10.3390/v18020173

**Published:** 2026-01-28

**Authors:** Jennifer Deborah Wuerth, Florian Ingo Schmidt

**Affiliations:** Institute of Innate Immunity, University Hospital Bonn, University of Bonn, 53127 Bonn, Germany; jen.wuerth@uni-bonn.de

**Keywords:** innate immune system, inflammasome, ASC speck, inflammation, virus, retrovirus

## Abstract

Inflammasomes are signaling complexes of the innate immune system that are assembled in distinct sentinel cell types to coordinate inflammation. As demonstrated by the emergence of viral antagonists and evasion mechanisms, inflammasomes are critical to contain viral infections. As virions are entirely composed of host cell-derived molecules, infection is either recognized by molecules or modifications exposed in unusual compartments, or by activities and host cell damage indicative of virus replication. Rather than enumerating all viruses that activate inflammasomes, this review classifies common pathways or signatures that activate antiviral inflammasomes. We define a set of minimal criteria that we think is critical to prove virus-triggered inflammasome assembly. We further discuss the consequences of virus-induced inflammasome assembly and define relevant open questions in the field.

Inflammasomes were identified more than 20 years ago as critical signaling complexes that coordinate inflammatory responses of the innate immune system. While initial work focused on inflammasome responses to sterile insults and bacterial infections, it is now increasingly clear that inflammasomes have also evolved to detect and counteract viral infections. This review aims to highlight common strategies applied to sense virus infections as well as the consequences of virus-triggered inflammasome assembly. We also seek to define minimal criteria for the experimental confirmation of virus-triggered inflammasomes to guide future studies. We include exciting new findings on inflammasome sensors such as NLRP1 and CARD8, which were previously poorly understood and respond to arboviruses and retroviruses, respectively. Due to the immense body of literature on virus-induced inflammasomes, we did not attempt to summarize every virus reported to activate this pathway but instead sought to derive common concepts from those experimental systems that allow a more detailed molecular understanding. In line with the topic of this Special Issue, the last chapter will focus on recent discoveries in the inflammasome activation by HIV-1.

## 1. Introduction—What Are Inflammasomes and How Do They Work?

The innate immune system of vertebrates has evolved a comprehensive network of sensors, signaling pathways, and effectors to counteract the threats of pathogens and other sources of damage or stress. Most somatic cell types can produce type I interferons to induce a generalized antiviral state in the tissue by autocrine and paracrine signaling [1]. In contrast, the inflammasome system employs a few sentinel cell types to rapidly initiate a strong pro-inflammatory response with short- and long-range effects on both antiviral states, influx of immune cells, and instruction of the adaptive immune system.

**Figure 1:**
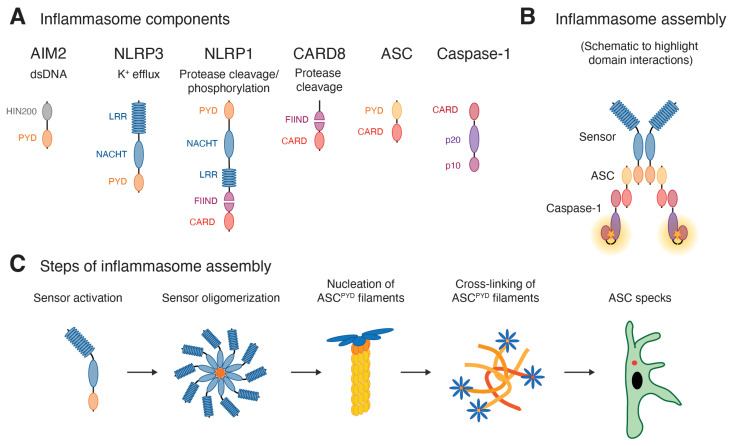
Antiviral inflammasomes. (**A**) The scheme represents the domain structures of inflammasome sensors known to detect viral infections, of the adaptor ASC, and of the effector caspase-1. (**B**) Schematic view of the domain interactions that give rise to a fully assembled canonical inflammasome (ignoring the true stoichiometry and 3D structure of the involved components). (**C**) In the cellular context, activation of inflammasomes involves the activation and oligomerization of the sensor (exemplified by a PYD-containing sensor; PYD in orange), the nucleation of ASC^PYD^ filaments (yellow), as well as the cross-linking of ASC^PYD^ filaments through ASC^CARD^:ASC^CARD^ interactions, which ultimately yield a macroscopic ASC speck (red) detectable by fluorescence microscopy and flow cytometry.

Inflammasomes are macromolecular signaling complexes that assemble in response to cytosolic evidence of infection or damage, and that coordinate the release of the potent pro-inflammatory cytokines IL-1β and IL-18 [2]. A limited number of sensor molecules converge by recruiting and nucleating the polymerization of the adaptor molecule ASC. ASC assembles into macroscopically visible structures termed ASC specks, which coordinate the activation of caspase-1. ASC specks are the prototypical outcome of canonical inflammasomes, here defined as complexes composed of a sensor, ASC, and caspase-1 (see Figure 1). ASC specks typically activate the pro-inflammatory protease caspase-1, which cleaves two classes of substrates: IL-1β and/or IL-18 are matured by proteolytic removal of the propeptide. Gasdermin D (GSDMD), in turn, is cleaved to unleash its pore-forming activity that in most cases kills the cell by pyroptosis. A few sensors can also directly engage caspase-1, while the related pro-inflammatory caspase-4 is directly activated by cytosolic LPS to form non-canonical inflammasomes. In both cases, caspase activity is more tuned towards GSDMD cleavage and pyroptosis rather than cytokine release, although human caspase-4 can also mature IL-18.

The different sensors capable of assembling inflammasomes have been reviewed in greater detail elsewhere [2] but will be briefly summarized here. All inflammasome sensors contain a Pyrin domain (PYD) or a caspase recruitment domain (CARD). Upon oligomerization of the sensor, small filamentous oligomers of the sensor PYD or CARD engage the adaptor molecule ASC, which features both a PYD and a CARD. AIM2 contains an N-terminal PYD and a C-terminal DNA-binding HIN200 domain and is consequently activated by cytosolic DNA. The eponymous sensor Pyrin comprises an N-terminal PYD, followed by a B-box-type zinc finger (BB), a coiled-coil domain (CC), and a C-terminal B30.2 domain. Pyrin inflammasomes are activated by distinct modifications of the actin cytoskeleton typical of bacterial infections. Most other inflammasomes belong to the NLR family and share an N-terminal CARD or PYD, a central NACHT and NAD domain, as well as C-terminal LRR domain. NLRP3 contains a PYD and is activated by diverse signals related to cell damage, in most cases involving the efflux of potassium ions. NLRC4 exhibits a CARD and is activated by another NLR, NAIP, once it directly binds bacterial type 3 secretion system components or flagellin. NLRP6 is activated by poorly defined signals related to disbalanced microbiomes (dysbiosis). NLRP1 features both an N-terminal PYD and a C-terminal CARD. The CARD is preceded by the function to find (FIIND) domain, which undergoes autoproteolytic cleavage to yield the ZU5 and UPA subdomains, which remain non-covalently associated. As detailed below, NLRP1 is activated by different viral, bacterial, or stress-related cues that all release the C-terminal UPA-CARD fragment that subsequently engages ASC.

In all cases, recruitment of ASC by different sensors initiates the polymerization of the ASC^PYD^ into filaments. ASC^PYD^ filaments are subsequently cross-linked through CARD:CARD interactions that result in spherical ASC specks, which control caspase-1 maturation and cytokine processing [3,4,5]. Importantly, ASC specks can also be revealed by antibody staining or fluorescent reporters as the most upstream readout for inflammasome assembly detectable by fluorescence microscopy or flow cytometry (see Section 2).

Lastly, the inflammasome sensor CARD8 has a domain structure that corresponds to the C-terminal end of NLRP1: an unstructured region is followed by an auto-processed FIIND and a C-terminal CARD. CARD8 is activated by viral proteases and—unlike most canonical inflammasome sensors—does not recruit the adaptor molecule ASC. Instead, it directly recruits and activates caspase-1 and thus likely resembles non-canonical caspase-4 inflammasomes in the arrangement of the signaling complex.

## 2. What Needs to Be Shown to Claim Canonical Inflammasome Activation?

Canonical inflammasome assembly can be assessed in many ways—not all of which are quantitative or equally definitive [6]. To demonstrate genuine activation of canonical inflammasomes by virus infection or any other trigger, we suggest adhering to the following guidelines. Of note, ASC-independent activation of caspase-1 or caspase-4 (or its paralog caspase-5) will require different readouts as pointed out below.

- **Quantification of the fraction of cells that assemble ASC specks** using immunofluorescence staining of ASC, or fluorescent reporters that incorporate fluorescent proteins (FP) into ASC specks (ASC-FP or caspase-1^CARD^-FP) by fluorescence microscopy or flow cytometry [7,8,9]. These experiments should be conducted in the presence of caspase-1 inhibitors such as VX-765 to avoid any effects caused by the loss of pyroptotic cells before analysis. For microscopy, this requires staining of nuclei or other markers to determine the number of cells. To allow robust conclusions, the number of cells should be sufficient to detect at least 100 (ideally 1000) specks in the absolute count in every biological replicate in the conditions that are interpreted as inflammasome assembly. It is critical to also quantify infection by staining of viral antigens or expression of virus-encoded reporter genes to determine the fraction of infected cells. Upregulation of inflammasome components at the level of transcription or translation is not indicative of inflammasome activation. If available, knockouts of inflammasome sensors and ASC should be included to identify the involved inflammasome sensor and validate ASC dependence. Identification of the responsible sensors by immunofluorescence staining with sensor-specific antibodies is prone to artifacts as ASC specks tend to non-specifically retain antibodies (Schmidt lab, unpublished observation; many publications lack the appropriate control). Such experiments should at the very least include controls that demonstrate that the respective antibody against an inflammasome sensor does not bind to specks that were assembled by a different inflammasome sensor. Ideally, quantification is conducted in cells that endogenously express the inflammasome sensor and ASC. Inflammasomes have been successfully reconstituted in HEK 293T cells by ectopically expressing ASC (or ASC-FP) as well as the respective sensor, allowing analysis by flow cytometry or microscopy [10,11]. One challenge of this setup is that ASC can oligomerize due to its mere overexpression in the absence of triggers, which is ideally minimized by the use of weak promoters and selected stable clones. Transcriptional regulation of genuine inflammasome components is in this case overruled by constitutive expression. Recruitment of caspase-1^CARD^-FP to ASC specks reports on both ASC speck formation and caspase-1 recruitment. As the levels of ASC remain unchanged and the reporter itself cannot form specks upon overexpression, this setup is less prone to background [8]. While cross-linking experiments may reveal ASC complexes by immunoblot [12], they do not yield any information on the fraction of cells that have assembled inflammasomes. Bulk experiments are also more vulnerable to loss of cellular material due to pyroptotic cell death.
- **Quantification of the concentrations of released IL-1β or IL-18** by ELISA, Homogeneous Time-Resolved Fluorescence (HTRF) [13], or other suitable assays to quantify the functional outcome of canonical inflammasome assembly. Detection of cleaved IL-1β or caspase-1 by immunoblot is less quantitative, as the actual response can be overestimated due to the precipitation of analytes from larger volumes. Control experiments should at the very least demonstrate that cytokine release is blocked by caspase-1 inhibitors and/or knockout. If knockouts are feasible, such experiments are ideally accompanied by the respective knockouts for the sensor and ASC. The material and methods should accurately report the number of used cells, the volume of the supernatant, and whether any measures to concentrate supernatants were applied.

To demonstrate that viral components contribute to inflammasome assembly, it is also critical to show inflammasome assembly in the context of infections (if necessary, with virus mutants that no longer interfere with inflammasome assembly). Mere overexpression of viral proteins may yield misleading results. Otherwise, the two criteria above are sufficient to prove canonical inflammasome assembly. In our opinion, additional (classical) bulk readouts described below are often redundant or less informative.

The following techniques can be used for screening purposes or to test additional conditions (drugs, knockouts, variants, kinetics) when inflammasome assembly has been established using the methods outlined above. They can also be used to quantify activation of CARD8 inflammasomes and non-canonical inflammasomes, which directly activate caspase-1 and caspase-4/5 in the absence of any ASC specks:

- **Quantification of ‘cell death’** via LDH release, dye uptake (and others): Cell death assays may not be sensitive and specific enough to detect inflammasome assembly in only a small fraction of cells. It is important to note that infection itself and other side effects of stimuli often cause cell death independent of inflammasomes. To distinguish this, genuine inflammasome-dependent cell death should be blocked by inhibitors of inflammatory caspases, which typically do not allow distinction of caspase-1 from caspase-4/5-dependent non-canonical inflammasome assembly. Ideally this is confirmed with the respective knockouts. It needs to be considered that LDH release assays quantify cell rupture and that GSDMD pores are not sufficient to release LDH tetramers [14]. Influx of DNA dyes such as propidium iodide (PI) may report on GSDMD pore formation (rapid response) or other biological processes that yield a loss of membrane potential, including late stages of apoptosis (delayed response). They are again meaningful if combined with caspase-1/-4/-5 inhibitors or knockouts.
- **Caspase-1 activity** [15]: Fluorogenic probes in which cleavage of a tetrapeptide (often YVAD or WEHD) de-quenches a fluorophore can be cleaved by active caspase-1 and other proteases [16]. However, active caspase-1 is rapidly lost from pyroptotic cells and fluorescent peptides do not enter cells in the absence of GSDMD pores [17,18]. Of note, peptide-based probes were designed under the assumption that caspase specificity is determined by the three amino acids before the aspartate residue found in all caspase cleavage sites [19]. However, caspase-1 and -4 have, e.g., been shown to recognize GSDMD using an additional exosite interaction, eliminating the need to match the previously defined consensus sequence around the aspartate [20,21]. Other assays designed for cell lysates and supernatants include a tetrapeptide that releases luciferin upon cleavage to allow luciferase-catalyzed bioluminescence [22]. In these assays, specificity has been gained by the addition of proteasome inhibitors. Experiments can be reliably interpreted if the respective caspase-1/-4/-5 inhibitors and/or knockouts are tested.
- **Detection of caspase-1/-4/-5, pro-IL-1β, and pro-IL-18 cleavage**: Detection of cleavage products of caspase-1 substrates by immunoblot allows the qualitative assessment of caspase-1 activity, where interestingly both mature caspase-1 and IL-1β are typically detected in the supernatant. This typically requires the concentration of supernatants and thereby offers the opportunity to substantially increase the sensitivity and detect minimal amounts of processed proteins. Such experiments should be accompanied by detailed information on cell numbers, volumes of the supernatant, and the fold-concentration of supernatants.

## 3. What Is Recognized by Inflammasomes During Viral Infection?

**Figure 2:**
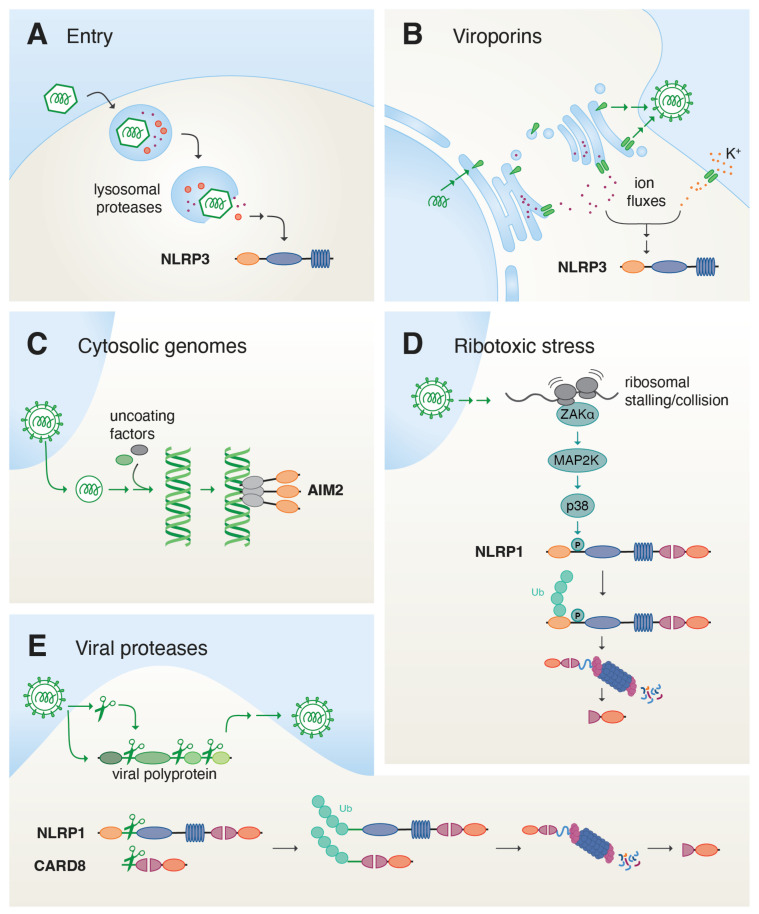
Activation of antiviral inflammasomes by different steps of the viral life cycle. Inflammasomes may be nucleated as a consequence of virus entry and the ensuing leakage of endo-lysosomal content (**A**), viroporin-mediated ion fluxes across different membranes (**B**), exposure of viral genomes (**C**), replication-mediated ribotoxic stress and the activation of MAP kinase signaling (**D**), as well as the activity of viral proteases that proteolytically cleave ‘tripwire proteins’ of the innate immune system (**E**).

The challenge of the innate immune system is to recognize rapidly evolving pathogens with a genetically determined set of pattern recognition receptors or sensors. Such receptors therefore often either recognize common pathogen-related patterns or molecules, or indirect evidence of pathogen activity in the cell. Unlike bacteria, fungi, and other parasites that contain unique biomolecules not found in the host cell, viruses are entirely constructed from host building blocks. This implies that the innate immune system requires additional information to recognize virus replication as a threat. One strategy is to exploit compartmentalization and recognize molecules that are not typically found in an organelle or cellular environment, including cytosolic dsDNA, dsRNA, or uncapped RNAs [23]. A second strategy is to recognize typical enzymatic or cellular activities associated with virus replication. In the following, we will examine the individual steps of virus replication in host cells to explore their potential to contribute to detection and inflammasome assembly (see Figure 2). Importantly, whether a given virus induces inflammasome assembly will not only depend on the detection by the respective sensors, but also on the tropism of the virus, i.e., whether virus components or viral activities will be exposed in inflammasome-competent cell types. Moreover, viruses will often also encode host-modulatory proteins that counteract inflammasome activation or its effectors. Finally, certain inflammasome responses depend on the upregulation of inflammasome components as part of the antiviral transcriptional response and can be inhibited indirectly by viral inhibition of host gene expression or translation.

### 3.1. Virus Entry

The first and major task of a virion is to deliver its genome to the cytosol or nucleus of a new target cell [24]. Virions are initially enriched on their target cells through interaction with attachment receptors, often using non-specific charge-based interactions. Subsequent binding to bona fide entry receptors may either trigger fusion of enveloped viruses with the plasma membrane or initiate endocytosis of enveloped and non-enveloped virions through a variety of different pathways. Cues in the endosomal compartment, including low pH, altered ion concentrations, or access to secondary receptors, subsequently allow the release of viral components to the cytosol. Enveloped virions typically fuse with the limiting membranes of early or late endosomes. Non-enveloped virions can either directly disrupt the entire endosome or form pores or other more transient gaps in the membrane to release virus substructures to the cytosol. Binding of some viral glycoproteins has been shown to induce pro-inflammatory gene expression [25] that can contribute to the expression of inflammasome components, i.e., priming. However, virus binding and endocytosis have not been shown to trigger inflammasome assembly to date. As viruses hijack common transport pathways for the uptake of nutrients or debris, the uptake of replication-competent virions per se can likely not be distinguished from other cellular uptake processes.

Disruption of lysosomes by crystals or the lysosomotropic drug LeuLeu-OMe potently activates NLRP3 inflammasomes, although the molecular mechanism is subject to controversy. Release of lysosomal cathepsins, induction of reactive oxygen species, or potassium have all been implicated [26]. Accordingly, the release of Adenovirus particles by lysosomal rupture activates NLRP3 inflammasomes as well [27]. It is likely that other non-enveloped virions induce similar responses, although the degree of membrane rupture may also define the exact outcome. It will further be interesting to test whether the reported activation of NLRP3 by altered endosomal functions will also contribute to detection of endocytosed viruses [28,29].

### 3.2. Uncoating

During endosomal escape or plasma membrane fusion, non-enveloped or enveloped virions either release viral genomes associated with proteins or deposit a viral capsid, i.e., an encapsidated form of the genome that is still shielded from further recognition. In the latter case, release of viral genomes to the site of replication therefore typically involves a distinct uncoating step. Protection of viral genomes within inaccessible capsids may shield them from recognition until the right compartment is reached, or until host-modulatory proteins are produced that counteract inflammasome assembly.

Cytosolic viral dsDNA genomes, for example, of poxviruses and herpesviruses, have been convincingly shown to activate AIM2 inflammasomes [30]. In many cell types, including human primary macrophages and keratinocytes, AIM2 was only sufficiently expressed after IFNγ treatment. It is possible that this avoids inappropriate AIM2 activation by DNA that is accidentally released from endosomes and mitochondria, or exposed during cell division. Poxviruses replicate in the cytosol of host cells and experiments with DNA replication inhibitors indicate that incoming poxvirus genomes were sufficient to activate AIM2, highlighting the sensitive detection machinery [31]. Despite the large size of viral genomes, several molecules of AIM2 need to come together in close proximity to nucleate ASC^PYD^ through short AIM2^PYD^ filaments that serve as seeds. ‘Dilution’ of AIM2 molecules on the long dsDNA seems to be avoided by a cooperative assembly mechanism of AIM2 on DNA, in which efficient DNA binding by the HIN domain (AIM2^HIN200^) relies on the ability of the AIM2^PYD^ to oligomerize [32].

Most other DNA viruses replicate in the nucleus to benefit from the existing host machinery for DNA replication, repair, and transcription. Those DNA viruses typically mask their genomes in a capsid during passage through the cytosol. The same is true for retroviruses, which reverse-transcribe their ssRNA genome into dsDNA in the incoming capsid [33]. DNA is either released from the capsid at the nuclear pore complex, as found for herpesviruses and adenoviruses, or after nuclear import of capsids, as observed for retroviruses, hepatitis B virus or parvoviruses [34]. Recognition of DNA virus infection by AIM2 relies on the accessibility of viral genomes, which may be the result of inefficient nuclear import or aberrant uncoating. It will be interesting to study whether effectors of the innate immune system help actively degrade capsids to prematurely expose the viral DNA in the cytosol, as proposed for detection of viral genomes by cGAS [35,36]. One such factor implied to catalyze premature uncoating of viral capsids is the GTPase MxB [37,38,39].

Numerous publications have reported that viral RNA genomes directly or indirectly stimulated inflammasome sensors, in most cases NLRP3 [40,41,42,43,44,45]. No common molecular mechanism has emerged, and how viral RNA genomes may trigger inflammasome assembly directly or indirectly will therefore require additional studies. This will most importantly rely on a better understanding of the molecular basis of NLRP3 activation. dsRNA of replicating alphaviruses, such as Semliki Forest Virus, has been postulated to directly activate NLRP1 [46], although later studies found that NLRP1 activation by dsRNA or RNA viruses required NLRP1 phosphorylation by p38 [47]. Whether dsRNA binding contributes to NLRP1 activation, or whether dsRNA sensing by other pathways is required to initiate p38 activation, will be the subject of future studies. Some RNA-binding proteins have also been proposed to nucleate novel inflammasomes by directly recruiting ASC, including RIG-I and MxA [29,48,49], but a genuine proof that these putative sensors can nucleate ASC polymerization is outstanding. Furthermore, viral RNA genomes are usually masked by viral nucleocapsid proteins and RNA replication is often restricted to viral replication sites that are partially surrounded by modified host membranes and may thus not be immediately accessible to RNA-binding molecules. An additional experimental complication is that RNA genomes are typically rapidly translated (in case of positive-stranded RNA viruses) or immediately transcribed into mRNA by co-packaged viral enzymes (negative-stranded RNA viruses). RNA exposure is therefore typically not temporally dissected from viral gene expression with its manifold consequences (see below).

### 3.3. Virus-Induced Ion Fluxes

Viruses modulate the cellular environment in many ways to facilitate virus replication and the production of progeny virions. Severral RNA viruses encode viroporins that insert into host cell membranes and allow passage of cations across the membrane along their electrochemical gradients. Coronavirus Orf3a and E, picornavirus 2B, hepatitis C virus (HCV) p7, influenza A virus (IAV) M2, and respiratory syncytial virus (RSV) SH pass H^+^, K^+^ or Ca^2+^ ions across membranes [50,51,52,53,54,55]. IAV M2 serves a dual role in the replication of IAV. During virus entry, M2 allows protons from the lumen of acidified endosomes to reach the interior of incoming virions, thus promoting disassembly of the matrix protein and uncoating [56,57]. In later stages of viral replication, H^+^ transfer from the lumen of the Golgi apparatus seems to impair the acidification of secretory vesicles and thereby prevents the premature acidification of the viral fusion protein hemagglutinin (HA) [58]. On top of that, several other viral proteins with additional functions, including the coronavirus glycoprotein E, or the HIV-1 auxiliary protein Vpu, have been shown or postulated to facilitate ion leakage through membranes [59,60]. While most viroporins are critically relevant for the assembly of progeny virus, their exact role in replication is in most cases poorly defined. An intriguing idea discussed for some viroporins is that the release of Ca^2+^ from extracellular and intracellular storage pools may initiate Ca^2+^-dependent processes.

Most viroporins have been shown to activate NLRP3 [51,52,54,59,61,62,63] and this is inherently linked to both the direct induction of ion fluxes as well as the indirect manipulation of ion gradients by affecting other ion transporters or channels. Ectopic overexpression of viroporins in the absence of virus infection may more drastically alter ion fluxes than infection itself, where expression and localization may be well-controlled to ensure the viability of host cells, which is critical for most phases of viral replication. To unambiguously link viroporin activity to inflammasome assembly, viroporin-mediated activation of inflammasomes should therefore also be verified in the context of virus infections. In case of IAV M2, specific inhibitors have been developed, which allow precise control over viroporin activity [58]. It is critical to ensure that viroporin inhibitors are applied in ways that do not simply block virus infection, as, e.g., M2-dependent uncoating of IAV needs to occur before any virus replication is possible.

### 3.4. Virus-Induced Stress Responses

#### 3.4.1. Ribotoxic Stress and Similar

Viral infection can affect the translation machinery at multiple levels, including the elongation of the growing peptide chain. Examples are the stress caused by alternative, suboptimal modes of translation (for example, the use of rare alternative codons or different termination signals) or viral targeting of the translation machinery to establish a host shut-off (such as the modification of translation elongation factors) [64]. Stalling and colliding ribosomes activate multiple responses to alleviate the translational stress. In case the underlying stress cannot be resolved, dysfunctional ribosomes drive the cell to apoptosis. Another outcome of impaired translation is the ribotoxic stress response. Here, the mitogen-activated protein (MAP) 3 kinase ZAKα is directly recruited to affected ribosomes and initiates further MAP kinase signaling via p38 and JNK [65,66,67]. ZAKα and p38 have recently been linked to the activation of the NLRP1 inflammasome by diverse stimuli [47,68,69,70]. This growing list of ribotoxic stress triggers that activate NLRP1 contains UV-B irradiation, which causes damage to ribosomal and messenger RNA, and multiple ribotoxins, which impair ribosome function. The latter includes multiple bacterial toxins such as diphtheria toxin, *Pseudomonas aeruginosa* exotoxin A and *Legionella pneumophila* sidL. Additionally, infection with Semliki Forest virus (SFV) and related alphaviruses appears to drive NLRP1 activation in a ZAKα- and p38-dependent manner [47]. While the link between alphavirus infection and the ribotoxic stress response is not yet clear, ZAKα and p38 appear to catalyze the phosphorylation of NLRP1 within its disordered linker region [47,70]. The latter potentially marks NLRP1 for subsequent ubiquitination and functional degradation of the N-terminus, resulting in increased availability of the C-terminal UPA-CARD fragment for inflammasome assembly and caspase-1 activation. The availability of reagents such as antibodies that selectively recognize specific phosphorylated forms of NLRP1 will aid the understanding of the role and chronology of post-translational modifications of NLRP1.

#### 3.4.2. Viral Proteases

Viral proteases are essential for the propagation of many positive-stranded RNA viruses, such as coronaviruses, picornaviruses and flaviviruses, as well as certain retroviruses. They release functional proteins from viral precursor polyproteins at multiple sequence-specific locations. Human NLRP1 and CARD8 make use of this and act as broad sensors of viral protease activities: a rapidly evolving, disordered N-terminal region in both NLRP1 and CARD8 mimics viral polyprotein cleavage sites and thereby acts as ‘tripwire’ for diverse viral proteases [71,72,73]. NLRP1 is cleaved by the 3C protease (3Cpro) of picornaviruses and 3CL protease (3CLpro) of coronaviruses such as SARS-CoV-2, while CARD8 is cleaved by HIV-1 protease [72,74,75,76,77]. The resulting cleavage of NLRP1 and CARD8 generates an unstable neo-N-terminus that is subsequently targeted for proteasomal degradation, liberating the UPA-CARD-containing C-terminus for inflammasome assembly and caspase-1 activation.

#### 3.4.3. Virus-Triggered Cell Death

Virus infection and the ensuing cell damage may likewise trigger cell death pathways that can indirectly be coupled to inflammasome activation in inflammasome-competent cell types. For example, induction of necroptosis may indirectly trigger potassium efflux and NLRP3 activation [78]. Likewise, activation of apoptosis has also been coupled to altered ion fluxes, including caspase-3-mediated activation of the ion channel pannexin-1, or caspase-3-mediated GSDME cleavage and pyroptotic cell death [79,80,81,82]. Necroptosis and apoptosis can be distinguished using small compound inhibitors of RIPK1/MLKL or caspase-3, respectively. Whether such indirect sensing of virus-triggered cell death is relevant for the activation of antiviral inflammasomes, and whether this is an irrevocable consequence of programmed cell death or requires distinct co-stimulation or regulation will be the subject of interesting future studies.

#### 3.4.4. Viral Protein Aggregates

Similarly to non-enveloped viruses, the uptake of viral protein aggregates into the endolysosomal system may result in inflammasome activation. The PB1-F2 protein of influenza A forms amyloid-like aggregates that resemble amyloid-like fibers. It has been reported that phagocytosis of these PB1-F2 aggregates results in the activation of the NLRP3 inflammasome [83,84,85]. Interestingly, PB1-F2 has also been proposed to inhibit NLRP3 activation when expressed intracellularly [83,86,87]. Both the activating and inhibitory activities of PB1-F2 differ between distinct influenza strains and further experiments are required to solve the discrepancies.

## 4. What Are the Molecular and Clinical Consequences of Inflammasome Activation During Viral Infection (And How Can We Measure Them)?

At the level of the infected cell itself, inflammasome-driven pyroptosis may serve to eliminate the viral replication niche and thereby restrict viral replication and spread throughout the host. If this is a relevant mechanism in any experimental system, an increase in the fraction of infected cells would be expected when pyroptosis is inhibited or when the involved inflammasome components are absent. Similarly, a rise in virus titer should be detectable when inflammasome responses are impaired. To achieve genuine restriction of viral replication by pyroptosis, however, multiple conditions need to be met: first, the induction of pyroptosis needs to precede the production and release of progeny virus. Second, pyroptosis would need to occur in the majority of infected cells and these cell types need to be critical for virus replication. In line with this, pyroptosis indeed appears to have a role in the depletion of HIV-infected cells, as detailed below. However, in our own work, we have frequently observed that the number of virus-infected cells significantly exceeds the number of inflammasome-assembling cells for various (rapidly replicating) RNA and DNA viruses, including Semliki Forest virus in keratinocytes, or vaccinia virus in macrophages [31,47].

Apart from the role of the pyogenic cytokines IL-1α and IL-1β in instructing local and systemic inflammation, these cytokines have also been reported to directly induce antiviral states in an auto- or paracrine manner, similar to interferons. Such a paracrine antiviral loop has indeed been observed between keratinocytes and fibroblasts [88]. In addition, the inflammasome-driven release of cytokines and DAMPs can also shape the adaptive immune response directly and indirectly [89]. Release of IL-1β mediates the expression of chemokines and adhesion molecules that favor influx of further immune cells into the tissue, which can indirectly promote adaptive immune responses [89,90]. IL-1β boosts the expression of costimulatory molecules on antigen-presenting cells and both IL-1β and IL-18 favor the differentiation of Th1 and Th17 cells and enhance T cell responses [91,92,93,94,95]. Additionally, pyroptosis can result in the release of antigens to the extracellular space and thereby contribute to cross-priming [96]. Finally, inflammasomes have recently been reported to induce the coordinated assembly of actin-rich filopodia that mark the pyroptotic corpse for antigen uptake and subsequent antigen presentation by dendritic cells [97].

The release of inflammasome-related IL-1β and IL-18 as well as other defined mediators in culture supernatants can be monitored via ELISA or HTRF. Mass spectrometry-based secretome analyses allow for an unbiased assessment of other released DAMPs and detection of those molecules for which specific ELISA or HTRF assays are not available. Antigen presentation as well as T and B cell activation (proliferation, cytokine release) can to some extent be reconstituted in vitro, but have mostly been studied in vivo.

As detailed above, direct antiviral contribution of pyroptosis and IL-1β can be assessed with straightforward methods. However, the contribution of inflammasomes to antiviral immunity and pathogenesis in vivo is still less well understood. In vivo, the systemic effects of antiviral inflammasomes are difficult to evaluate in both animals and patients, as inflammasome-related cytokines, especially IL-1β, are hard to detect in peripheral blood. Cytokines only rise very transiently or may be locally restricted [98,99]. A potential solution is to detect longer-lasting antagonists, such as IL-1RA, which are upregulated by IL-1β, as an indirect readout for inflammasome activation [100,101,102]. Additionally, viruses—in contrast to chemically defined inflammasome activators—simultaneously trigger multiple innate immune pathways that may converge, compete, or drive secondary activation as discussed above. Most conclusions have been drawn from experiments with knockout mice for inflammasome components. However, it is not trivial to distinguish direct from indirect effects. Finally, differences in host receptors, tissue architecture, restriction factors, and the general immune response cause inter-species differences in the infection process and pathogenesis. For example, functional differences exist between human and murine NLRP3 [103,104], while human and murine NLRP1 strongly differ in their domain structure and their expression levels in keratinocytes [105]. CARD8 is not encoded by mice at all [73]. Consequently, addressing the role of inflammasome activation for viral infection at the organism level is often hampered by the lack of suitable tools and model systems.

Regardless of the distinction of indirect and direct effects, a protective early role of inflammasomes has been clearly described for influenza A virus infections [41,106,107]. The existence of viral evasion and antagonism of inflammasome activation, downstream signaling, pyroptosis and IL-1β activity argues for a role of inflammasomes in the antiviral immune response. For example, poxviruses encode putative NLRP1 inhibitors, PYD-containing ASC inhibitors, pan-caspase inhibitors, as well as soluble IL-1β and IL-18 receptors [108,109,110,111]; Herpes viruses encode direct inhibitors of the AIM inflammasome [112], while HSV-1 ICP0 has been proposed to antagonize NLRP1 activation [113]. RNA viruses likewise encode antagonists of inflammasome activation, including influenza A virus NS1 [114], SARS-CoV-2 NSP1 and NSP13 [115], and paramyxovirus V proteins [116,117].

In contrast, uncontrolled inflammasome activation and excessive cytokine and DAMP release drive hyperinflammation. The prolonged presence of inflammatory mediators can drive cytokine storms, and the increased influx of inflammatory cells can cause tissue damage and thus exacerbate pathogenesis [41,106,107,118]. For example, high levels of IL-1β, IL-18 and cleaved caspase-1 in the sera or lungs of COVID-19 and influenza patients were associated with severe disease and poor clinical outcome [119,120]. High IL-1β levels were detected in patients with severe dengue infection and a role for IL-1β in vascular leakage and tissue injury was shown in a corresponding mouse model [121,122,123]. Furthermore, NLRP3 inhibition or deficiency in mouse models of RSV infection decreased lung immunopathology as well as RSV-induced allergy exacerbation [123].

The protective or pathogenic contribution of specific cytokines at the tissue or organism level can be evaluated using specific cytokine antibodies (e.g., Canakinumab), receptor-neutralizing biologics (e.g., anakinra) or knockouts of cytokines or receptors. However, it needs to be kept in mind that bioactive IL-1α can be released upon multiple forms of cell death or damage. Thus, IL-1 receptor-dependent effects are not necessarily due to inflammasomes and require further controls.

Similar to the antiviral interferon response, the timing and duration of the inflammasome response may determine whether antiviral and beneficial effects prevail, or whether harmful and pathologic consequences dominate the host response. For instance, early inflammasome activation during influenza A virus infection appears to contribute to viral clearance but becomes harmful in later stages of infection by driving hyperinflammation in mouse models [41,106,107,118]. IL-1β and IL-18 as well as other inflammatory cytokines, influx of immune cells into the lungs and lethality were reduced in infected mice deficient for NLRP3, ASC or caspase-1.

The number of cells susceptible to virus infection can be high. Extensive pyroptosis and the associated cytokine and DAMP release can lead to harmful tissue damage. A low fraction of cells that assemble inflammasomes and undergo pyroptosis upon infection may therefore represent a trade-off between the antiviral response and the necessity to limit tissue damage. The need for tight regulation of inflammasome activation and pyroptosis is also reflected in the ongoing adaptation between host and pathogens: The sequences (and function) of inflammasome components and their regulators vary between species. Genes have been duplicated or lost throughout evolution. This is accompanied by strong positive selection of certain inflammasome components or specific subdomains. One example is the strong positive selection of the ‘tripwire’ regions of NLRP1 and CARD8, which selectively acquired certain viral protease cleavage sites in the primate but not other mammalian lineages [71,72].

Inflammasomes have important roles in viral infection and are promising targets for ameliorating hyperinflammation-driven pathology. However, therapeutically targeting inflammasomes in patients demands a detailed understanding of both their distinct roles during the different stages of infection as well as the infection process within the individual patient.

## 5. Inflammasome Responses to HIV-1

In 2024, 40.8 million people were living with HIV, 1.3 million were newly infected, and 630,000 HIV-related deaths were recorded, making HIV/acquired immunodeficiency syndrome (AIDS) a considerable burden to public health [124]. Progressive depletion of CD4^+^ T cells and chronic inflammation drive disease progression to AIDS [125,126]. The establishment of a latent virus reservoir in resting CD4^+^ T cells and tissue macrophages shortly after infection and its persistence despite antiretroviral therapy is a major roadblock in eradicating HIV [127,128,129,130,131].

HIV virions bind to cell surface receptors and fuse with the plasma membrane to deliver viral capsids into the cytosol. Within the capsid, the packaged viral reverse transcriptase transcribes the viral RNA into double-stranded DNA. Viral DNA genomes are imported into the nucleus, released from the capsid, and integrated into the host genome by the packaged viral integrase. During the late phase of HIV replication, viral genes are transcribed and the viral mRNAs for Gag and Gag-Pol precursor polyproteins, the Env glycoproteins as well as regulatory and accessory proteins are exported from the nucleus. The Env glycoproteins are translated at the rough endoplasmic reticulum and traffic to the plasma membrane via the secretory pathway. In contrast, the Gag and Gag-Pol polyproteins are translated in the cytoplasm. Gag and Gag-Pol also traffic to the plasma membrane, where new virions are assembled from the viral genomic RNA, Gag, Gag-Pol and Env, and subsequently bud off. Importantly, budding is followed by particle maturation, which is crucial for infectivity: the viral protease (HIV^PR^) within Gag-Pol is activated and, in its dimeric form, cleaves Gag and Gag-Pol. Gag is cleaved into matrix, capsid, nucleoprotein, p6 and two spacer peptides, whereas Gag-Pol processing additionally yields the viral reverse transcriptase, integrase and protease. The processing of the precursor proteins is accompanied by marked changes in virion structure, yielding the conical capsid core which contains and protects the viral RNA, nucleocapsid protein, as well as reverse transcriptase and integrase.

Inflammasome-associated cytokines IL-1β and IL-18 are increased in the plasma of HIV patients [132,133,134,135]. Furthermore, persistently elevated IL-18 levels were found to be associated with the failure of highly active anti-retroviral treatment. Similarly, higher levels of IL-18 in the plasma of HIV progressors compared to controllers correlated with lower CD4^+^ T cell count and higher viral load [134,136]. High plasma levels of IL-1β and IL-18 drive the differentiation of naïve CD4^+^ T cells to Th1 and Th17 cells and enhance T cell responses [54,55,56,57,58]. The depletion of Th17 cells then further contributes to compromised gut epithelial barriers. The resulting circulation of microbial ligands fuels chronic immune activation [95].

While monocytes with ASC specks are detectable in the blood of HIV patients [137], the role of the inflammasome response in myeloid cells in the pathogenesis of HIV/AIDS is not entirely clear. In contrast, the progressive depletion of infected T cells is a major characteristic of HIV and driver of pathogenesis. Thus, we will mainly focus on the inflammasome response in HIV-infected T cells.

Productively infected and activated T cells die by caspase-3-driven apoptosis [138,139]. Yet, this only accounts for a small fraction of depleted T cells. Instead, one of the major hallmarks of HIV-1 infections is the massive loss of quiescent lymphoid T cells that succumb to caspase-1-mediated pyroptosis [138,140]. Caspase-1 inhibition limits the loss of CD4^+^ T cells in both human lymphoid aggregate cultures (HLAC) and in mouse models with a humanized immune system, suggesting a role for inflammasome activation in HIV pathogenesis [140,141,142]. IFI16 was initially proposed to assemble a DNA-dependent inflammasome upon abortive infection of CD4^+^ T cells and thereby trigger pyroptosis [143]. However, while the presented data supported a role of IFI16 in pyroptosis, direct evidence of IFI16-nucleated inflammasomes was not presented. For example, the presence of ASC specks was not evaluated. Furthermore, Bosso et al. later observed that ASC specks are only nucleated by AIM2 but not by the other three human PYHIN proteins IFI16, MNDA and PYHIN1, whereas IFI16 was found to act as restriction factor by interfering with recruitment of transcription factor Sp1 [144,145]. While Monroe et al. rule out NLRP3 as responsible inflammasome sensor in lymphoid T cells, another report proposed a role for NLRP3 in HIV-driven pyroptosis of peripheral CD4^+^ T cells of patients with HIV [146].

While it appeared difficult to reconcile these studies despite differences in experimental systems and virus strains, another inflammasome sensor entered the stage: HIV-1 protease (HIV-1^PR^) cleaves CARD8 in its unstructured ‘tripwire’ region and thereby activates it, resembling the activation of NLRP1 by picorna- or coronavirus proteases [147,148,149]. Interestingly, the cleavage site in human CARD8 is not conserved or functional in other primates, whereas human CARD8 can also be cleaved by the proteases of SIV strains of chimpanzees and rhesus macaques [148,149]. Furthermore, HIV-1 strains that are resistant to protease inhibitors cleave and activate CARD8 with varying efficiency [147,149,150], suggesting ongoing evolutionary conflicts and adaptation. As CARD8 is not expressed in murine cells, its function needs to be studied in cells reconstituted with human CARD8, or even better in human cell lines or primary cells that express endogenous levels of the inflammasome sensor. In vivo experiments to evaluate CARD8 function can only be conducted in humanized mice [151].

CARD8 is expressed in unstimulated and activated blood CD4^+^ T cells, as well as naïve and memory CD4^+^ and CD8^+^ T cells in lymphoid tissues. In line with the direct recruitment of caspase-1 by CARD8, CARD8 activation in resting CD4^+^ and CD8^+^ T cells drives pyroptosis, but not cytokine release. In contrast, activated CD4^+^ T cells are not responsive to CARD8 activation [147,148,152,153,154]. HIV infection thus drives rapid loss of peripheral blood- and tonsil-derived quiescent CD4^+^ T cells, which is recapitulated in transferred T cells in a humanized mouse model [147,148]. CARD8 is also activated by HIV^PR^ in monocytes and monocyte-derived macrophages [149,150]. IFI16 was not required for the loss of CD4^+^ T cells in the studies by Wang et al. [147,148] and, contrary to Zhang et al. [146], these studies suggest that inflammasome activation is largely (but not entirely) independent of NLRP3 and ASC [148,150]. Taken together, CARD8 appears to be the dominant inflammasome sensor of HIV-1 infection.

Although HIV^PR^ is essential to produce infectious progeny virus, its activity is tightly regulated and the protease usually remains inactive until viral budding, as premature or delayed activation blunts infectivity [155,156,157,158]. Nonetheless, the amount of HIV-1^PR^ that is delivered by incoming virions during viral entry is sufficient for CARD8 activation and pyroptosis of CD4^+^ T cells [147]. Productive infection is not required. Similarly, the packaged HIV-1^PR^ in incoming virions is also sufficient for CARD8 activation in acutely infected monocytes and monocyte-derived macrophages [149,150].

Of note, HIV^PR^ activity in infected cells can also be artificially triggered prematurely: non-nucleoside reverse transcriptase inhibitors (NNRTI), such as rilpivirine and efavirenz, enforce cytosolic dimerization of the Gag-Pol polyprotein and thereby activate HIV^PR^. This way, NNRTI treatment can also drive CARD8 activation in infected CD4^+^ T cells as well as monocytes and macrophages. Importantly, a combination of latency reversal agents (LRAs) to reactivate viral gene expression and enforced HIV^PR^ activation by NNRTI treatment, can drive latently infected T cells into pyroptosis, resulting in the clearance of this viral reservoir [147,148,149,150,151]. Consequently, a combination of LRAs and NNRTIs, on the one hand, and CARD8 sensitization via DPP9 inhibition, on the other hand, has been suggested as potential therapeutic approach to clear latently infected T cells and macrophages and thus eliminate viral reservoirs [148,151]. If successful, this might present a long-awaited breakthrough in the treatment of patients with HIV. However, in contrast to T cells, in which CARD8 activation primarily drives pyroptotic cell death, monocytes and macrophages also release mature IL-1β and thereby contribute to inflammation. Thus, a potential role of CARD8-driven inflammatory cytokine release from these cell types in chronic inflammation and HIV/AIDS pathogenesis has to be considered. Further investigation to avoid the risk of excessive tissue inflammation is thus required.

## 6. Conclusion, Outlook, Open Questions, Current Challenges to Overcome

Inflammasome sensing, activation and effects remain understudied for a wide range of viruses relevant to human disease. In many cases, the genuine trigger to activate inflammasome sensors in virus-infected cells remains unidentified. Similarly, the contribution of inflammasomes to host immunity and viral pathogenesis is poorly defined, likely due to a lack of tools to specifically quantify IL-1β responses in the tissue.

The contribution of inflammasomes to the pathogenesis of viral infection makes them attractive targets for therapeutic intervention. As one promising example, CARD8 activation with a combination of NNRTI treatment and DPP9 sensitization might be exploited to eliminate the HIV-1 reservoir in latently infected T cells in the treatment of HIV. Yet, the clinical applications of such strategies will require a more detailed mechanistic understanding of both the sensor biology in target cells and the virus-induced cues that initiate inflammation.

In our opinion, the following open questions require further attention:

1. Which cell types can assemble (antiviral) inflammasomes?
2. How are viral intermediates distinguished from cellular components?
3. Which co-stimulatory cues are required to license inflammasome components?
4. Which common strategies of viral antagonism are emerging?
5. Which consequences of inflammasome assembly and pyroptosis are most relevant to counteract infections? Which outcomes contribute to pathogenesis?
6. What are the immediate consequences of IL-1β signaling and which cells sense it?
7. How are antiviral inflammasomes and cell-autonomous responses such as interferon responses integrated and coordinated (between cell types and signaling pathways)?
8. How can human-specific inflammasome responses be studied at a systemic or tissue-wide level to assess their contribution to antiviral responses?
9. How are virus-specific adaptive immune responses shaped by inflammasomes?

## Data Availability

No new data were created or analyzed in this study. Data sharing is not applicable to this article.

## Abbreviations

The following abbreviations are used in this manuscript:

AIDS	Acquired immunodeficiency syndrome
AIM2	Absent in melanoma 2
ASC	Apoptosis-associated speck-like protein containing a CARD
BB	B-box-type zinc finger
CARD	Caspase recruitment domains
CARD8	Caspase recruitment domain-containing protein 8
CC	Coiled-coil domain
cGAS	cyclic GMP-AMP synthase
DNA	Deoxyribonucleic acid
ELISA	Enzyme-linked immunosorbent assay
FIIND	Function to find domain
FP	Fluorescent protein
GSDMD	Gasdermin D
HIV-1	Human immunodeficiency virus 1
HLAC	Human lymphoid aggregate culture
HTRF	Homogeneous time-resolved fluorescence
IAV	Influenza A virus
IL-1β	Interleukin-1 beta
LDH	Lactate dehydrogenase
LRA	Latency reversal agents
LRR	Leucine-rich repeat
MAP	Mitogen-activated protein
MLKL	Mixed lineage kinase domain-like pseudokinase
NACHT	NAIP, CIITA, HET-E and TP1
NLRP1	NACHT, LRR and PYD domains-containing protein 1
NLRP3	NACHT, LRR and PYD domains-containing protein 3
NNRTI	Non-nucleoside reverse transcriptase inhibitors
PR	Protease
PYD	Pyrin Domain
RNA	Ribonucleic acid
RIPK1	Receptor-interacting serine/threonine-protein kinase 1
RSV	Respiratory syncytial virus
SFV	Semliki Forest virus
